# Non-Inferiority of Point-of-Care Ultrasound Compared to Radiography to Diagnose Upper Extremity Fractures in Children

**DOI:** 10.3390/children9101496

**Published:** 2022-09-30

**Authors:** David Troxler, Carlos Sanchez, Thierry de Trey, Johannes Mayr, Michael Walther

**Affiliations:** 1 Interdisciplinary Pediatric Emergency Unit, University Children’s Hospital Basel, 4057 Basel, Switzerland; 2 Pediatric Research Center, University Children’s Hospital Basel, 4057 Basel, Switzerland; 3 Department of Pediatric Surgery, University Children’s Hospital Basel, 4057 Basel, Switzerland

**Keywords:** child, fracture, pain, radiography, ultrasound, POCUS

## Abstract

Conventional X-ray imaging for fracture diagnosis is time-consuming and exposes patients to ionizing radiation. Additionally, the positioning of the injured limb for standardized X-ray imaging is painful. Point-of-care ultrasound (POCUS) is increasingly available in medical offices and emergency rooms. This study aimed to prove the non-inferiority of POCUS compared to X-ray imaging with respect to diagnostic sensitivity, pain, and investigation time in the diagnosis of long-bone fractures of the upper extremity in children. Children and adolescents (1–18 years old) presenting to the UKBB emergency service between May 2020 and May 2021 with suspected upper extremity fracture were included in the study. Before obtaining X-ray images, we conducted a POCUS examination of the injured limb. Pain scores at inclusion as well as maximum pain scores during X-ray and ultrasound examinations were documented. The duration of POCUS and X-ray examinations was compared. We examined 403 children with POCUS and plain X-ray imaging. The mean age (±SD) of the children was 10.6 (±3.5) years. The non-inferiority of POCUS compared to X-ray was confirmed with an estimated sensitivity of 0.95 and a lower confidence interval of 0.93. Maximum pain during POCUS was significantly lower compared to pain at inclusion (*p* = 0.002) or maximum pain during radiographic examination (*p* = 0.03). POCUS examination took 3.9 (±2.9) min in the mean whilst the mean duration for obtaining the X-ray images was 16 (±37) min (*p* < 0.001). POCUS for diagnosing upper extremity fractures in children proved as sensitive as standard X-ray imaging and was significantly faster and less painful. Future prospective studies are required to confirm our findings.

## 1. Introduction

Fractures of the upper extremities are common in children. Approximately 200 children with suspected upper extremity fracture are treated in the pediatric emergency room (pER) of our university children’s hospital each month.

If a fracture is suspected in a child presenting to our pER, two separate X-ray images have to be obtained to diagnose or exclude a fracture, according to the current standard operating procedure (SOP) [1]. This procedure has several disadvantages. X-ray imaging exposes children to ionizing radiation. Depending on the suspected fracture, the injured limb needs to be positioned differently for the two separate planes of the X-ray images, resulting in pain. Furthermore, X-ray imaging is time-consuming, as the work routine of the attending physician is interrupted once the child is sent to the radiologic imaging facility and then returns to the pER after the procedure. The time between requesting radiographic examination and interpreting the image is called therapeutic turnaround time or brain-to-brain time [2].

X-ray images are interpreted by the pER pediatrician, and treatment is based upon this interpretation. A secondary reading by a pediatric radiologist for quality control is only performed later on. This procedure has been adopted by several hospitals internationally [3]. The accuracy of X-ray interpretation by pER pediatricians relative to that achieved by pediatric radiologists amounted to approx. 90% in several studies [4,5,6], with figures ranging from 84% to 99% [7,8].

Ultrasound diagnosis of pediatric fractures in different body regions has been studied for several years [9]—in particular, for the ribs and sternum [10]. In the past few years, the ultrasound diagnosis of upper-extremity fractures in adults and children has been studied [11,12,13,14,15,16,17,18]. A recent validation study in Germany conducted in five study centers included a total of 498 children [19]. A recent meta-analysis [20] showed a good overall sensitivity and specificity, but only three studies [21,22,23] included more than 200 patients. A new position paper published by the German Society of Orthopedics and Traumatology states that ultrasound might be able to replace X-ray imaging in the future in a substantial number of injured children [24].

Ultrasound has several advantages over X-ray imaging. It does not expose patients to ionizing radiation, devices are mobile, and the examination can be performed in the child’s preferred position of the injured limb with minimal movement of the affected limb, thus causing less pain. Point-of-care ultrasound (POCUS) is readily available, and it can be applied directly by a pER physician. However, evidence for its effectiveness is still limited, and its application in routine care is uncommon.

In this prospective non-inferiority study, we aimed to establish whether it is safe to use ultrasound instead of X-ray imaging to screen for suspected long-bone fractures in children presenting to the pER with trauma to the upper extremity.

## 2. Materials and Methods

### 2.1. Study Design

After obtaining ethical board approval (EKNZ 2020-00032, 18 March 2020), we conducted the study at the pER of the University Children’s Hospital of Basel (UKBB). We included children with a clinical suspicion of a long-bone fracture of the upper extremity who presented to our pER over a period of 53 weeks.

Physicians (attending pediatric physicians and advanced residents) with or without previous fracture ultrasound experience were trained in a 1 h theory and hands-on course or had to prove equivalent education. Standardized ultrasound examinations and documentation procedures (standardization of imaging planes, minimum number of documented images during each examination) were defined for the proximal humerus (3 imaging planes: longitudinal anterior, lateral, and posterior), elbow joint region (1 imaging plane: longitudinal posterior to detect a fat pad), distal forearm (6 imaging planes: longitudinal dorsal, volar, and lateral of radius and ulna), metacarpal bones (2 imaging planes: longitudinal dorsal and volar), and phalanx of the fingers (2–3 imaging planes: longitudinal dorsal and volar as well as lateral on dig I, II, and V). Examiners were free to add additional planes.

### 2.2. Sample Size Calculation

Hypothetical study populations with nx patients with fractures were simulated counting up from n1 = 1, n2 = 2, n3 = 3, ... to n_lowest_. The n_lowest_ was defined as the lowest n_x_ for which the lower end of the 95%-confidence interval (95%-CI) of POCUS sensitivity did not cross the non-inferiority margin (λ) of 90% in 80% of 10,000 repetitions. As suggested by the International Conference on Harmonisation (ICH) of Technical Requirements for Registration of Pharmaceuticals for Human Use), 95%-CI for non-inferiority trials can be chosen as one-sided tests [25]. The lowest n_x_ fulfilling these requirements was n = 189, i.e., approx. 189 patients with fractures were required for our study. The sample size n, including patients without fractures, was calculated by dividing the total number of patients with fractures (n = 189) by the assumed proportion of included patients with fractures (%positive) multiplied by 100. Hence, the total number of patients to be included in the study was 189/50 × 100, i.e., n = 378.

### 2.3. Inclusion/Exclusion Criteria

Children aged 0–18 years with trauma to the upper extremities and suspected fracture of a long-bone were eligible for the study. Mean age (±SD) of the children was 10.6 (±3.5) years. Patients were included only if the pER workflow permitted their study inclusion and a study investigator was available to perform the POCUS procedure.

Exclusion criteria were any of the following: patient needing immediate medical attention (Australasian Triage Score: score 1 or 2) [26]; severely displaced or open fractures; fractures complicated by neurovascular compromise distally to the injured region; patients for whom imaging studies were obtained before the ER visit; patients with a recent history of prior fracture of the injured area; patients with known allergy to ultrasound gel; patient with suspected ‘battered child’ syndrome; or unavailability of a study investigator able to perform the POCUS examination within a reasonable time frame (15 min).

### 2.4. Study Procedures

After we had obtained written informed consent from the parents or caregivers of the child, patients underwent POCUS examination. A total of 69 examinations were conducted using a Zonare z.one ultra with a linear probe (L14-5W). The remaining 334 (83%) POCUS examinations involved a SAMSUNG HS60 device with a linear probe (LA3-14AD).

The examiners documented their ultrasound findings in a case report form, stating clearly whether they would have recommended confirmation of the diagnosis by conventional X-ray examination in the routine care setting. In the current study setting, X-ray images were obtained for every patient. Subsequently, patients underwent treatment according to the radiographic diagnosis.

Pain was evaluated at inclusion as well as after POCUS and X-ray examinations by rating the maximum pain experienced by the child during the examination and at rest afterwards. Pain levels were monitored regularly during the study, and any pain increase from baseline of 2/10 points or more was recorded in a safety report.

Pain was rated on a scale of 0 to 10 by a registered pediatric emergency nurse using “Kindliche Unbehagens- und Schmerz-Skala” KUSS [27] for children below the age of 4 years, Faces Pain Score—Revised (FPS-R) for children aged between 4 and 11 years, and Numeric Rating Scale (NRS) for children aged 12 years and older [28]. Pain medication was administered according to the hospital protocol for routine clinical care.

### 2.5. Primary and Secondary Outcomes

The primary outcome was proof of non-inferiority of POCUS to detect upper extremity long-bone fractures compared to X-ray imaging.

The secondary outcomes included comparison of pain scores recorded at different time points of POCUS and X-ray imaging. In addition, we compared the time periods required to conduct POCUS and X-ray examinations of the injured region of the upper extremity.

### 2.6. Data Collection and Statistical Analysis

The examiners entered the POCUS data and pain scores in a paper case report form (CRF). We scanned the CRFs immediately after completion and mailed them to the study administration center. No changes were possible afterwards.

X-ray diagnostic findings were extracted from the picture archiving and communication system (PACS). Radiologists were blinded with respect to POCUS findings. Paper CRFs were entered into Securitrial electronic CRFs by the primary investigator. Statistical analysis was conducted using R [29].

Non-inferiority was calculated with a one-sided CI. We assumed that the sensitivity of both ultrasound and X-ray images was 95%, and the proportion of included patients with fractures %_positive_ = 50%. δ was set at 5%. Hence, the non-inferiority margin was sensitivity_X-ray_ − δ, i.e., 95% − 5% = 90%. Non-inferiority was confirmed if the lower end of the one-sided 95%-CI did not lie below the non-inferiority margin of 90%. Analysis was based on the full analysis set. R function BinomCI from the package DescTools was used to calculate the 95%-Cis using the Agresti-Coull interval method [30]. For this analysis, all examinations with inconclusive results for which examiners required X-ray confirmation (n = 87) were counted as positive.

Differences in pain and duration associated with POCOS and X-ray imaging were calculated using two-sided paired *t*-test.

## 3. Results

In total, 439 patients were eligible for the study, but 11 patients refused to participate. Thus, 428 patients were included in this study. For 25 (5.8%) patients, the documentation of the ultrasound examination was not scanned before the X-ray was completed; therefore, they were excluded from the analysis. Overall, 403 patients were analyzed for the primary outcome. Table 1 shows the patient demographics and baseline variables as well as the fracture sites.

In total, 14 investigators were involved in the study. Among these, 4 conducted fewer than 10 examinations, 5 completed between 10 and 20 examinations, and 5 conducted more than 20 examinations. One investigator completed 194 (48%) examinations.

### 3.1. Primary Outcome

**Figure 1:**
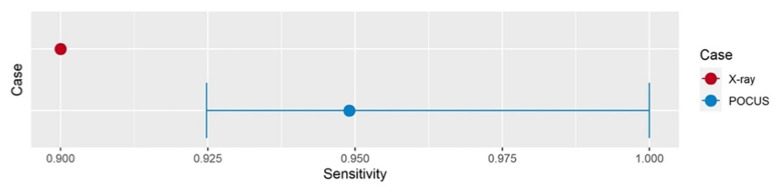
One-sided confidence interval of POCUS vs. X-ray imaging.

Assuming 90% sensitivity for fracture diagnosis, non-inferiority of POCUS vs. radiographic imaging in detecting upper extremity fractures was confirmed with an estimated sensitivity of 0.95 and a lower confidence interval of 0.93 (Figure 1).

For this analysis, all patients for whom the POCUS examiners stated that they would request confirmatory X-ray examination because of diagnostic uncertainty (n = 87) were considered positive. The sensitivity was thus calculated by dividing the number of patients for whom fractures were diagnosed correctly (n = 210) by all patients with fractures diagnosed by X-ray imaging (n = 220).

In total, 183 confirmatory X-ray examinations excluded a fracture. For 120 (65.6%) of these, the examiners stated after POCUS that they would be confident enough to rely solely on their ultrasound examination and would be comfortable to discharge patients without confirmatory X-ray.

In a subset analysis excluding the 194 examinations conducted by one investigator, non-inferiority was also confirmed with an estimated sensitivity of 0.97 and a lower confidence interval of 0.94 (n = 209).

### 3.2. Secondary Outcomes

**Figure 2:**
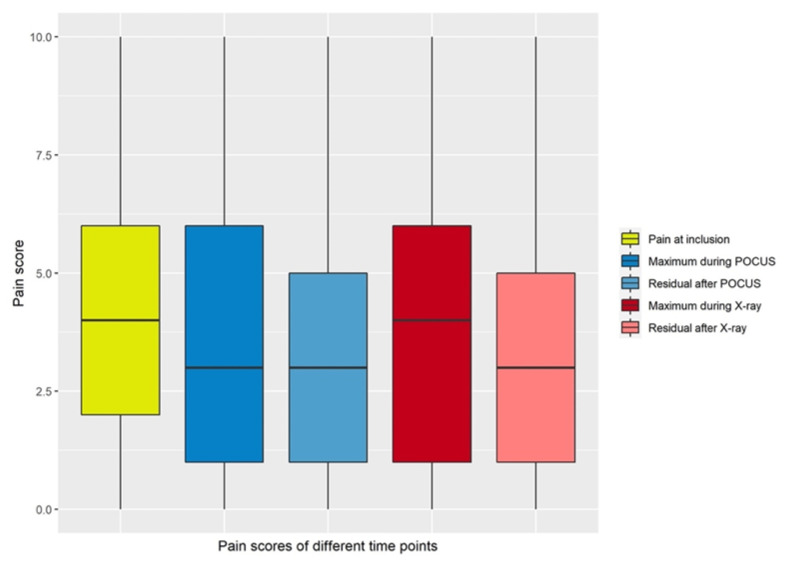
Box plots of pain scores observed at different time points.

**Figure 3:**
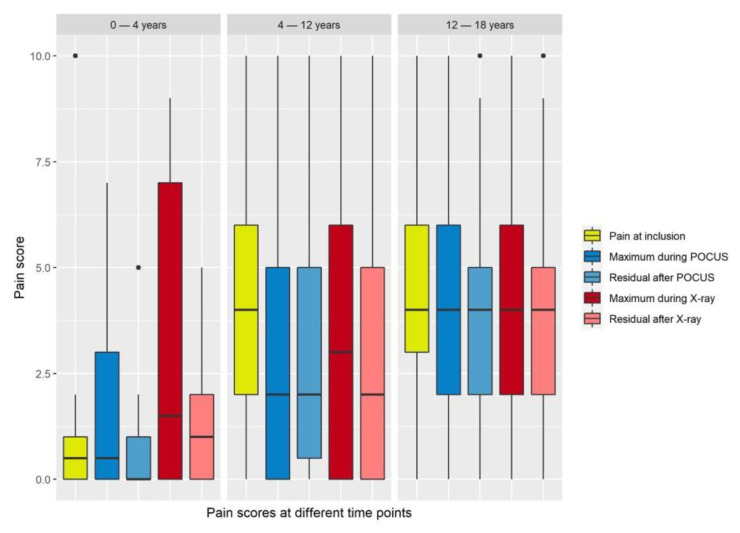
Box plots of pain levels by age group and rating scale: 0–4 years: KUSS; 4–12 years: VAS; 12–18 years: NRS.

Mean maximum pain scores during ultrasound examinations were significantly lower than mean pain scores at inclusion or during X-ray examinations (Figure 2 and Figure 3, Table 2).

Maximum pain during POCUS was significantly lower compared to pain at inclusion (*p* = 0.002) or maximum pain during radiographic examination (*p* = 0.03).

For this analysis, 11 incomplete datasets with 1 or more missing pain values were excluded (n = 392).

The time required to complete the POCUS examination compared to the time span elapsed between ordering and completing the X-ray images is shown in Table 3. POCUS examination took 3.9 (±2.9) min in the mean, whilst the mean duration for obtaining the X-ray images was 16 (±37) min (*p* < 0.001).

**Figure 4:**
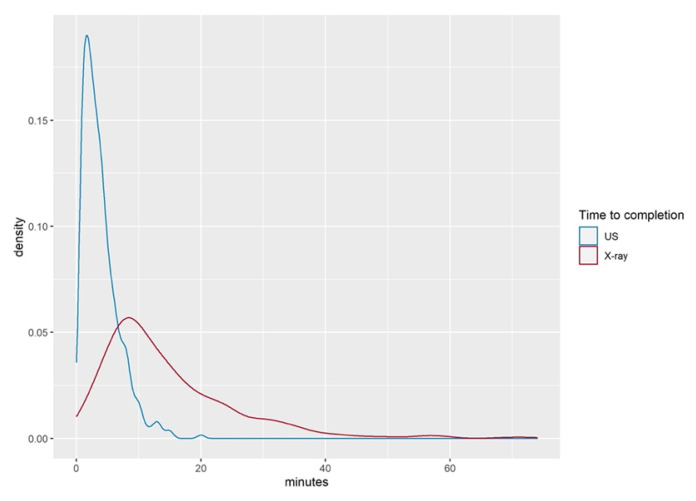
Time difference between ultrasound investigation and radiographic image study.

Figure 4 shows the time difference of POCUS and radiographic imaging examinations.

One patient arrived after midnight and underwent X-ray imaging the next day because no “non-urgent” X-ray was available at our facility between 11 p.m. and 7 a.m.

### 3.3. Learning Curve for POCUS

**Figure 5:**
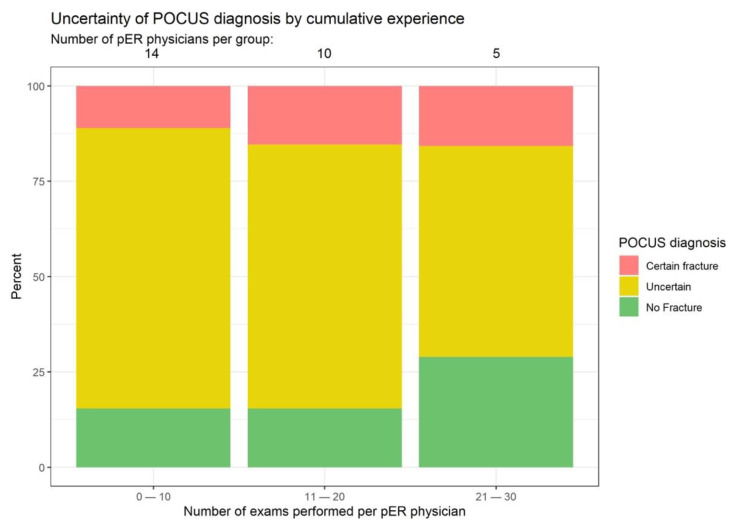
Learning progression. Red: positive fracture sign in POCUS and X-ray without subjective need for an X-ray; green: exclusion of fracture in POCUS and X-ray without subjective need for an X-ray; yellow: results with subjective need for an X-ray.

Diagnostic uncertainty among POCUS examiners declined over time, and pER physicians became increasingly confident with ultrasound diagnosis, thus creating less need for X-ray confirmation. Already after 20 examinations, the examiners declared that they were able to exclude fractures exclusively based on POCUS in approx. 25% of patients (Figure 5).

## 4. Discussion

The present study confirmed that long-bone fractures of the upper extremity in children can be as reliably excluded by POCUS as by X-ray already after the short POCUS training of the examiners. By allowing examiners to opt for an X-ray confirmation of their diagnosis based on POCUS in cases of uncertainty, we provide a safe framework even for POCUS beginners.

Additionally, we were able to prove that there was no pain increase due to POCUS examination. As pain medication was dispensed according to the hospital protocol, we expected to see a progressive decrease in pain. The fact that the initial pain was reduced significantly during POCUS examination may be attributed in part to the cooling effect of the ultrasound gel applied at room temperature.

We hypothesize that the pain increase seen during the X-ray examination was probably caused by the painful positioning of the injured extremity. In contrast, painful positioning maneuvers were avoided during the POCUS examination.

The mean duration of the POCUS procedure was <4 min (measured from POCUS start to last image). Additionally, POCUS examiners became faster and more competent with increasing experience.

Ultrasound imaging is more operator-dependent than X-ray imaging, computed tomography (CT), or magnetic resonance imaging (MRI). Ultrasound images obtained in different planes and at variable settings may be interpreted differently by investigators. This may make pER physicians reluctant to use ultrasonic scanning in fear of facing legal consequences from divergent image interpretation.

### Study Limitations and Strengths

Because as many as 48% of all POCUS examinations were completed by a single examiner, our study results and learning-curve data cannot be generalized. Only one study examiner was a full-time pER physician, while all other examiners were part-time physicians, thus explaining the heterogeneous numbers of examinations conducted.

Moreover, several possible fracture locations were rare in our study, thus reducing the validity of our findings for such rare upper-extremity fractures. Thus, adequately powered studies for specific fracture locations are clearly needed.

In terms of study strengths, this adequately powered, prospective study may help to reduce the number of plain X-ray images needed to diagnose injuries of the upper extremity, thus minimizing the use of ionizing radiation in children.

## 5. Conclusions

By demonstrating non-inferior fracture exclusion by POCUS compared to X-ray imaging, we were able to adopt an algorithm allowing us to treat patients without fractures in the absence of confirmatory X-ray imaging. However, examiners must be aware of the limitations of POCUS examination. Ultrasound is an operator-dependent examination and involves subjective assessment. Fracture diagnosis based on POCUS must therefore be compatible with the clinical picture. The reliability of identifying fractures with residual stability and minor clinical relevance (buckle fractures) as well as those with no potential for displacement needs to be investigated in a future prospective study.

The accurate determination of POCUS training time needed for practitioners to provide precise fracture diagnosis should be addressed in a multicenter study involving larger groups of examiners. However, allowing the POCUS operator to opt for confirmatory X-ray investigation if needed assures a safe setting for both the patient and the POCUS examiner.

## Figures and Tables

**Table 1:** Baseline patient characteristics (n = 403).

Gender n, (%)	male 237 (59)	female 166 (41)
Age (years)	mean 10.6	min 1, max 17.9
Side n, (%)	left 196 (49))	right 207 (51)
Site of injury n, (%)	elbow (positive fat-pad sign)	54 (13)
	humerus	10 (3)
	metacarpal bone	47 (12)
	phalanx	90 (22)
	forearm	202 (50)
Final diagnosis n, (%)	fractured 220 (55)	not fractured 183 (45)

**Table 2:** Pain levels measured at different time points.

	Time Point	Pain Score (IQR)
Mean pain score (1st/3rd interquartile range; IQR)	inclusion	3.8 (2/6)
	max_US_	3.4 (1/6)
	after_US_	3.2 (1/5)
	max_X-ray_	3.6 (1/6)
	after_X-ray_	3.1 (1/5)
Pain initial vs. Pain max US	difference 0.39	*p* = 0.0018
Pain max_X-ray_ vs. Pain max US	0.26	*p* = 0.03

US = ultrasound.

**Table 3:** Duration of POCUS and X-ray imaging.

US duration mean (1st/3rd quartile; minutes)	3.9	(2/5)
X-ray duration (1st/3rd quartile; minutes)	16	(7/19)
Mean difference US vs. X-ray time (minutes)	12.5	*p* < 0.001

US = ultrasound.

## Data Availability

Not applicable.
